# LncRNAs, the Molecules Involved in Communications With Colorectal Cancer Stem Cells

**DOI:** 10.3389/fonc.2022.811374

**Published:** 2022-01-27

**Authors:** Boyang Fan, Qian Zhang, Ning Wang, Guiyu Wang

**Affiliations:** ^1^ Department of Colorectal Cancer Surgery, The Second Affiliated Hospital of Harbin Medical University, Harbin, China; ^2^ Department of Colorectal Surgery, Cancer Hospital of the University of Chinese Academy of Sciences (Zhejiang Cancer Hospital), Hangzhou, China; ^3^ Department of Pharmacology (State-Province Key Laboratories of Biomedicine-Pharmaceutics of China, Key Laboratory of Cardiovascular Medicine Research, Ministry of Education), College of Pharmacy, Harbin Medical University, Harbin, China

**Keywords:** colorectal cancer, cancer stem cell, lncRNA, chemoresistance, metastasis, signal pathway

## Abstract

Colorectal cancer stem cells (CRCSCs) can actively self-renew, as well as having multidirectional differentiation and tumor regeneration abilities. Because the high functional activities of CRCSCs are associated with low cure rates in patients with colorectal cancer, efforts have sought to determine the function and regulatory mechanisms of CRCSCs. To date, however, the potential regulatory mechanisms of CRCSCs remain incompletely understood. Many non-coding genes are involved in tumor invasion and spread through their regulation of CRCSCs, with long non-coding RNAs (lncRNAs) being important non-coding RNAs. LncRNAs may be involved in the colorectal cancer development and drug resistance through their regulation of CRCSCs. This review systematically evaluates the latest research on the ability of lncRNAs to regulate CRCSC signaling pathways and the involvement of these lncRNAs in colorectal cancer promotion and suppression. The regulatory network of lncRNAs in the CRCSC signaling pathway has been determined. Further analysis of the potential clinical applications of lncRNAs as novel clinical diagnostic and prognostic biomarkers and therapeutic targets for colorectal cancer may provide new ideas and protocols for the prevention and treatment of colorectal cancer.

## Introduction

Colorectal cancer (CRC) is the third leading cause of cancer-related deaths worldwide, with its high recurrence and metastasis rates being the main reasons for poor prognosis (1, 2). Colorectal cancer originates from the colorectal mucosal epithelium and glands, and the development and progression of CRC is a multi-step, multi-stage and multi-gene process, involving the progression from hyperplasia to adenoma to carcinoma. This process includes the transformation of colonic epithelial cells into adenocarcinoma cells resulting from genetic and epigenetic instability; and the remodeling of the surrounding stromal tumor microenvironment (3).

Tumors are diseases that involve cell proliferation and differentiation. The biological characteristics of tumors include continuous cell proliferation and poor differentiation, as well as the ability to invade and metastasize to other sites (4). Cancer stem cells (CSCs), an important component of the tumor microenvironment, constitute a small subpopulation of cancer cells (5). CSCs are a class of tumor cells that initiate and maintain tumor growth with stem cell-like property, or “stemness”, involving self-renewal and multidirectional differentiation, and participate in tumorigenesis, progression, recurrence, metastasis and resistance to chemotherapy (6). Stemness is not only considered an inherent cellular property is also as a property of cell populations that are highly dependent on environmental conditions (7).

Long non-coding RNAs (lncRNAs), a class of related genomic regulators that are involved in a wide range of biological processes, play an important role in the origin and malignant progression of tumors. LncRNAs can function in cis, near their own transcription sites, or in trans, at distant genomic or cellular locations (8). Various lncRNAs have been shown to regulate gene expression at multiple levels, including epigenetic, transcriptional, and post-transcriptional regulation (9). Multiple lncRNAs bring other regulatory molecules (e.g., mRNAs, miRNAs, and DNA) into close proximity with one another and with proteins (e.g., chromatin modifying complexes, transcription factors, E3 ligases, and RNA-binding proteins (RBPs)), essentially creating a flexible molecular scaffold that fosters the chemical interactions required to maintain cellular activity (10).

Many studies have been performed to determine the biological relevance of lncRNAs to CSCs. To date, lncRNAs have been found to be involved in the regulation of multiple functions of CSCs, possibly by regulating key factors of multiple pathways, such as transcription factors, miRNAs, exosomes, and cell modifying enzymes (11). This review provides a comprehensive analysis of lncRNAs that regulate CRCSCs and their mechanisms of action. Analysis of these regulatory activities of lncRNAs may provide basic principles for the study of the molecular mechanisms of CRCs and new clinical treatment strategies targeting CRCSCs with lncRNAs.

## Colorectal Cancer and Cancer Stem Cells

### Pathogenesis and Influencing Factors of Colorectal Cancer

Two hypotheses have been advanced regarding tumor origin and the mechanism of tumorigenesis, the “clonal evolution” hypothesis theory and the “cancer stem cell” hypothesis. Traditionally, tumorigenesis is regarded as a process of clonal evolution, involving the accumulation and evolution of multi-stage mutations in somatic cells over about 10-15 years (12). CRC is a representative tumor characterized by the accumulation of mutations, including the activation of oncogenes, such as KRAS, MYC, and EGFR; the inactivation of anti-cancer genes, such as APC, DCC, and TP53; mutations in mismatch repair genes, such as MLH1, MSH2, PMS1, and PMS2; and the overexpression genes such as PTGS2 and CD44 (13). However, the “clonal evolution” hypothesis cannot satisfactorily explain the mechanism of tumorigenesis.

The “cancer stem cell” hypothesis has therefore been advanced to explain tumorigenesis. This hypothesis suggests that CSCs are the only populations in tumor tissue of tumor origin and propagation, and that these cells maintain the malignant phenotype of tumor cells (14). The first CSCs in gastrointestinal tumors were identified in 2007 in CRC. Moreover, CSCs were found to play a variety of roles in the development and progression of CRC (15, 16).

The “cancer stem cell” and “clonal evolution” hypotheses, however, are not mutually exclusive. CSCs are the cells of origin during the initial stage of tumorigenesis. During tumor progression, CSCs accumulate mutations and are subjected to clonal evolutionary effects. This results in the continuous differentiation of CSCs into tumor cells with stronger invasive and metastatic properties and a greater selective growth advantage, with these cells becoming the dominant cell population, eventually resulting in tumor formation (17). Both mechanisms therefore operate during tumor development and progression, with their main dominant roles shifting over time and space, ultimately determining the characteristic changes at different stages of tumor development.

### Functions and Characteristics of Cancer Stem Cells

CSCs can originate from three main sources: 1) normal intestinal stem cells (ISCs) that undergo oncogenic transformation to produce CSCs; 2) progenitor cell dedifferentiation into cells with more stem cell-like characteristics; 3) and self-renewal of CSCs (4).

Human CRCSCs, located in colonic crypts, were originally isolated based on their expression of CD133. These cells have been shown to induce tumors similar to primary malignancies in mice (15, 16). CSCs are dynamic, rather than static, populations that are subject to constant change due to multiple external and intrinsic factors, and may vary in number and phenotype during tumor progression. Therefore, tumor expression of CSC markers should not be considered a general property of the tumor, but rather a property that can change over time. CRCSC markers identified to date include EphB2^high^ (18), EpCAM^high^/CD44^+^/CD166^+^ (19), CD133^+^ (15, 16), CD26^+^ (20), ALDH^+^ (21), LGR5^+^ (22) and CD44v6^+^ (23, 24). The ideal markers for CSCs are those required to maintain their stemness. However, the surface markers of CRCSCs identified to date are also expressed by normal ISCs, indicating that these surface markers cannot easily distinguish CSCs from tumor cells, thus limiting the clinical application of surface markers as potential therapeutic targets.

CSCs have multiple biological properties, including heterogeneity and plasticity. Tumor heterogeneity is key to the high recurrence rate and refractoriness to treatment of common tumors. Tumor heterogeneity may be intra- or inter-tumor, in that different patients with the same pathological type of tumor may have different genotypes, or multiple primary tumors of the same type in an individual may have different genotypes (25). Inter-tumor heterogeneity mainly depends on the regulation of gene expression and methylation, whereas intra-tumor heterogeneity is mainly due to multiple gene mutations (26). The phenotypic and functional heterogeneity of CSCs are thought to be associated with the high recurrence rate and heterogeneity of cancers (27). Tumor heterogeneity can be evaluated by single-cell sequencing.

The plasticity of CSCs refers to their ability to switch between different functional states, including quiescent/proliferative, drug-sensitive/resistant, symmetric/asymmetric division, epithelial-mesenchymal transition/mesenchymal-epithelial transition, and stem/non-stem states. CSC plasticity plays an important role in tumor progression, metastasis and chemoresistance, facilitating the adaptation of these cells to their changing microenvironment (28). Both tumor heterogeneity and plasticity are driven by a combination of genetic, epigenetic, and microenvironmental factors that together lead to functional diversity at the intertumor, intratumor, and subclonal levels. The plasticity of CSCs can complicate clinical treatment, with the number of CSCs in CRC and other tumors significantly increasing after chemotherapy or radiation treatment. Treatment with cytotoxic antitumor agents, which initially target proliferating CSCs, may result in the partial selective survival of quiescent CSCs, with multiple cycles of treatment enhancing CSC proliferation and self-renewal (29, 30). Targeted CSC treatment may also enhance CSC regeneration, as non-stem cells have the ability to reconstitute CSCs. Targeted therapies may also induce a reactive response, leading to the resurgence of more aggressive tumors. During or after treatment, stresses on microenvironmental signaling and/or mutations in drug-resistant genes may give rise to CSCs with new functional properties. The interconversion of CSCs and transitionally expanded progenitor cells suggests that more aggressive clones may be randomly selected at both levels (7). Regeneration of CSCs after treatment may be inhibited by combining CSC-targeted therapy with drugs that inhibit microenvironmental or epigenetic mechanisms. Thus, interfering with tumor cell plasticity may provide new strategies for maintaining the activity of conventional and targeted anticancer drugs.

### Regulation of Colorectal Cancer by Cancer Stem Cells

CSCs have been found to regulate pathophysiological processes during various stages of CRC, including the regulation of metastasis (31). Metastasis is a major cause of CRC-related deaths and determines overall disease survival and prognosis. The epithelial mesenchymal transition (EMT) is an important pathological process associated with tumor metastasis and is closely related to the acquisition and maintenance of CSC properties and drug resistance. EMT is thought to transform epithelial cells into mesenchymal cells with stem cell-like properties and convert non-CSCs into CSCs. Thus, tumor cells that undergo EMT can acquire the properties of CSCs (32–34). CSCs spread and metastasize after EMT, and become circulating tumor cells (CTC) with stem cell-like properties, while maintaining their self-renewal capacity, heterogeneity acquired from asymmetric division, and plasticity to adapt to new environments. This connection suggests that heterotypic signals that trigger EMT, such as those released by activated inflammatory substrates, may also be important for the creation and maintenance of CSCs.

CSCs are also involved in the mechanism of CRC resistance to drugs. Cancer cells with EMT characteristics were found to be resistant to chemotherapy, and cancer cells resistant to drugs were found to have EMT characteristics. Drug resistance mechanisms associated with CSCs mainly include: 1) enhanced ability to repair damaged DNA, 2) high expression of ABC transports and ALDH enzymes that promote drug efflux, 3) increased expression of anti-apoptosis proteins, and 4) a resting and slow proliferation due to being in G0 phase (35). Conventional anti-cancer therapies cannot eradicate CSCs, and drug pressure induces more rapid gene mutations in CSCs, often increasing their number and enabling them to eventually adapt to the selection pressures imposed by treatment, leading to cancer recurrence and further drug resistance. However, it is worth noting that not all drug-resistant cells are CSCs, as tumors have specific resistance mechanisms against each drug, not necessarily through stemness pathways.

### Factors Influencing the Function of Cancer Stem Cells

The functional properties of CRCSCs are influenced and regulated by multiple pathways. Exogenous signaling molecular systems from the stem cell microenvironment and endogenous stem cell factors (e.g., transcription and epigenetic factors) integrate with each other to coordinate stem cell proliferation, suppress the expression of key differentiation genes, maintain the undifferentiated state of stem cells, and regulate the differentiation potential and progression of stem cells through multiple signaling networks and interactions. Transcription factors involved in regulating the undifferentiated state of stem cells include Oct4, Sox2, Nanog, KLF4, and STAT3 (36–40), and signaling pathways involved in regulating the homeostasis of CSCs include the Wnt, Notch, Hedgehog and TGF-β/BMP pathways (41–44). Ongoing research on the crosstalk among oncogenic developmental signaling pathways mentioned above and between these pathways and other oncogenic pathways (e.g. the TNF-NF-κB, KRAS-RAF-MAPK, PI3K-AKT-mTOR and BCR-ABL1 cascades) has hinted at their profoundly complex roles in cancer. Further determination of molecular crosstalk is important for the development of therapeutic strategies targeting CSCs and may uncover opportunities to inhibit multiple cascades by directly targeting one. Notably, cancer cells and CSCs share certain signaling pathways. For example, Wnt pathway members play a prominent role in the regulation of CRC cells and malignant stem cells. These cells differ in their levels of Wnt pathway activation, with CRC cells showing widespread overactivation of the Wnt pathway in colorectal cancer cells, whereas most CSCs are in a quiescent state, with the Wnt pathway being enhanced (41). These findings suggest that a single signaling pathway can activate different downstream target genes in various cell types. In addition, pathway activation can be positively or negatively influenced by other pathways, providing further evidence for the complexity of intra- and extracellular signaling networks.

Mounting evidence has indicated crosstalk between the tumor and numerous components of its microenvironment, including stromal cells, the tumor microvasculature, extracellular matrix (ECM), and the hypoxic microenvironment. A dynamic microenvironment is critical to maintain the balance of stem cell self-renewal and differentiation, as well as the stability of the stem cell population. Stemness arises from the continuous adaptation of cancer cell populations to microenvironmental signals. Abnormal activation of signals or structural alterations in the CSC microenvironment are important stimuli that induce a malignant phenotype in CSCs.

Non-coding RNAs have been reported to have multiple regulatory effects on the activity of CSCs, and likely constitute targets for clinical applications, such as diagnosis and treatment. Cancer cells with high metastatic potential or resistance to treatment have been reported to communicate with neighboring cells through cell-to-cell mediators, called exosomes, enabling the primary microenvironment to facilitate the initiation of metastasis or inducing resistance in cells previously sensitive to drugs (45, 46). LncRNAs can also participate in intracellular and intercellular communications and signaling, a role also known as extracellular microenvironmental remodeling processes. These lncRNAs may be packaged into exosomes and transferred to recipient cells, resulting in the dissemination of stemnesss, which leads to homogenization and uniformity among tumor cells. Future studies will likely assess the mechanism by which the microenvironment influences CSC dynamics and functions, possibly exposing weak points in CSC survival strategy that could be exploited therapeutically.

## Biological Role of LncRNAs

LncRNAs are a class of RNAs longer than 200 nt that are not involved in encoding proteins and are located in the nucleus or cytoplasm of cells. LncRNAs, which have many different origins, are classified as sense, antisense, bidirectional, intronic, and intergenic lncRNAs, based on their position on the genome relative to protein-encoding genes. LncRNAs were initially thought to be by-products of genomic transcription without biological function. They were not recognized as an important new transcript category until large-scale sequencing of the mouse full-length cDNA library (47). Recent studies have shown that lncRNAs are able to regulate gene expression at the epigenetic, transcriptional, post-transcriptional, and translational levels, and that their functions and mechanisms are related to their genomic and intracellular localization (9, 10).

### LncRNAs in the Cytoplasm

Cytoplasmic lncRNAs exert their biological roles mainly through two pathways. 1) utilizing complementary base pairing, cytoplasmic lncRNAs act as molecular sponges to adsorb miRNAs through a competitive endogenous RNA mechanism (ceRNA), thereby regulating the expression of downstream target genes at the post-transcriptional level. 2) antisense lncRNAs are involved in the post-transcriptional or translational regulation of genes by pair-binding to mRNAs and affecting their translation, splicing and stability. For example, in prostate cancer, the lncRNA ARLNC1 interacts with AR mRNA to regulate its cytoplasmic level, and LincRNA-p21 directly binds to JUNB and CTNNB1 transcripts to repress the translation of both (48, 49).

### LncRNAs in the Nucleus

LncRNAs in the nucleus can regulate target gene expression at the transcriptional level by binding transcription factors to the promoter regions of target genes. Depending on the relative locations of the lncRNAs and target genes in the genome, lncRNAs located on the same chromosome as their target gene are called cis-regulating, whereas lncRNAs located on a different chromosome as their targets are called trans-regulating (8). In addition, lncRNAs in the nucleus can recruit chromatin remodeling complexes that alter chromatin modifications and structure (e.g. DNA methylation, histone methylation or acetylation modification) and epigenetically regulate the expression of target genes (50). LncRNAs have also been found to interact directly with DNA to form RNA-DNA triplex structures (51).

### LncRNAs in the Cytoplasm and Nucleus

LncRNAs in the cytoplasm and nucleus can act as molecular scaffolds for complexes, bind to regulatory proteins, such as RNA binding protein (RBP), influence the formation of protein polymers, and regulate the activity and localization of proteins.

Many lncRNAs not only have sequence features but also have higher structural domains (motifs) that provide a basis for predicting their biological functions. Genetic modification models such as systemic knockout or overexpression, conditional knockout or overexpression of lncRNAs in model animals are critical evidence for studying the function of lncRNAs.

## Research Progress on LncRNAs That Participate in the Regulation of CRCSCs

LncRNAs are expressed in high abundance in CRCSCs, making their abundance a prerequisite for their wide range of regulatory roles. This section describes the ability of lncRNAs to regulate CRCSCs, as well as their mechanisms of action.

### LncRNAs Regulate CRCSCs Through ceRNA Mechanism

LncRNAs have been shown to act as ceRNAs for specific microRNAs, thus regulating the expression of their downstream target genes in the cytoplasm (9, 10). For example, LINC00511 was found to be significantly upregulated in CRC tissues and cells, acting as an oncogene and upregulating NFIA through sponge adsorption of miR-29c-3p. NFIA has oncogenic effects in many human tumors, promoting cell proliferation, metastasis and stemness, thereby accelerating the development of CRC (52, 53). The regulatory roles of the stemness-related proteins Sox-2, Oct-4, CD44 and Nanog on CSCs were evaluated by observing their change processes (54). Lin28 is a highly conserved RNA-binding oncoprotein that promotes CRC progression and metastasis by upregulating stem cell-related genes and/or by activating the Wnt signaling pathway (55, 56). The lncRNA PVT1-214 was shown to mediate the up-regulation of Lin28 mRNA through miR-128, increasing the expression and stabilization of Lin28 protein and enhancing the proliferation, invasion and stemness of CRC cells. Upregulated Lin28 mRNA was also found to suppress the expression of let-7, a classical oncogenic miRNA targeting RAS expression, further promoting CRC development (57–59). The DIS3 like 3’-5’ exoribonuclease 2 (DIS3L2), which is closely associated with the Lin28/let-7 pathway in several cancers, was shown to be downregulated by knockdown of lncRNA AC105461.1, which is located upstream of the DIS3L2 promoter, thereby enhancing the stemness of CRC (60, 61). The enhancer regions of DIS3L2 and its antisense transcript AC105461.1 share several transcription factors, suggesting that the binding of AC105461.1 to DIS3L2 mRNA affects the stability or promoter activity of the latter, thereby regulating its expression.

CSCs also play important roles in racial differences in CRC. Tumorigenesis and progression are two important processes in tumor biology, and many epithelial tissue tumors, including CRCs, are generated from a small fraction of CSCs through oncogenic transformation. LncRNAs not only regulate tumor progression through CSCs, but also promote tumorigenesis. CSCs are mainly enriched in colonic mucosal epithelial cells, and overexpression of miR-1207-5p in normal colonic epithelial cells promotes the production of CSCs. As a host gene for miR-1207-5p, PVT1, a lncRNA upregulated in the colon mucosae of African Americans, increases the proportion of CSCs by enhancing miR-1207-5p expression and may contribute to the higher incidence of CRC in African Americans than in whites (62). Sox2 was found to bind to the PVT1 promoter and enhance its transcription in breast cancer, suggesting that a similar positive feedback loop in CRC may promote its stemness profile (63).

Utilizing the same ceRNA mechanism, additional lncRNAs were identified in CSCs, including the lncRNA HOXD-AS1, which mediates the upregulation of astrocyte elevated gene-1 (AEG-1) and enhancer of zeste homolog 2 (EZH2) through miR-217. The lncRNA HOXD-AS1, which was shown to promote tumor progression and to be related to poor prognosis in patients with CRC, may be clinically applied as an indicator of prognosis in these patients (64). CASC21 was shown to enhance the expression of human growth hormone 1 (HGH1) by recruiting the transcription factor POU class 5 homeobox 1B (POU5F1B) in the nucleus and sponging miR-485-5p in the cytoplasm, thereby promoting CRC stemness (65). Branched chain amino acid transaminase 1 (BCAT1), which is overexpressed in a variety of tumors and promotes tumor progression, is upregulated by TMPO-AS1 through the targeting miR-98-5p, which in turn promotes CRC cell stemness (66). DPP10-AS1 was shown to inhibit the proliferation of CRCSCs by regulating miR-127-3p and adenylate cyclase 1 (ADCY1), and to exert a tumor suppressor function in CRC (67). DNA-damage-inducible transcript 4 (DDIT4) and sulfatase 1 (SULF1) have now been shown to be associated with several types of cancer (68, 69). TPTEP1 was observed to be high expression in CRCSC-enriched spheroids. The expression levels of DDIT4 and SULF1 were significantly positively correlated with TPTEP1, and significantly negatively correlated with miR-148b-3p. According to the predicted binding site, these correlations may be explained by mRNA-miRNA network (70).

The expression of key proteins that regulate CSCs, including STAT3, Oct4, Sox2, Nanog, Sox9, and MSI1, is, in turn, regulated by lncRNAs, thereby participating in process by which CSCs regulate CRC development and progression. STAT3 is an important transcription factor involved in regulating the undifferentiated state of stem cells. The lncRNA BCAR4 was shown to promote CRC stemness by targeting the miR-665/STAT3 signaling pathway (71). Similarly, MALAT1 was found to mediate stem cell-like properties and cellular glucose metabolism in human CRC cells through regulation of the miR-20b-5p/Oct4 axis (72). The oncogenic lncRNA FARSA-AS1 (transcript antisense RNA) activated by the stemness transcription factor Sox9 was found to upregulate Sox9 expression through its uptake of miR-18b-5p, forming a positive feedback loop. By binding miR-28-5p, Sox9 increases FARSA mRNA levels, thus promoting CRC growth, stemness and metastasis (73). The RNA-binding protein MSI1 is a stemness factor important for regulating the proliferation and differentiation of stem cells and precursor cells (74). For example, LINC01567 (LOCCS) was found to enhance the proliferation of CRCSCs and CRC tumorigenesis by targeting microRNA-93 to upregulate MSI1 (75).

ZEB1 has been shown to enhance BMI-1, Sox-2, and KLF-4 expression by targeting the miR-200 family, which in turn promotes stemness and tumorigenicity of CSCs (76, 77). The lncRNA XIST was found to promote EMT, stemness, and metastasis of CRC by competing for miR-200b-3p, thereby upregulating ZEB1 expression (78). The lncRNA UICLM promotes CRC stemness, growth and liver metastasis by targeting miR-215 to upregulate ZEB2 expression (79). LINC00657 promotes invasion of CRCSCs by mediating the upregulation of ZEB1, ZEB2 and Snail2 expression through miR-203a (80).

Hypoxia in the stem cell niche is important in maintaining the undifferentiated phenotype of normal stem cells and CSCs (81). HIF-2α is a key hypoxia-inducible factor in this process and can interact with various CSC-related pathways, including c-Myc and Oct4, to regulate stem cell proliferation, differentiation and pluripotency (82, 83). The lncRNA-HIF2PUT, a transcript upstream of the HIF-2α promoter, has been found to enhance these properties of CRCSCs by upregulating the expression of HIF-2α (84). The lncRNA AK000053 is a novel hypoxia-inducible that affects the expression of calmodulin CDH1, transcription factor ZEB1, SALL4 and BMI1 through downregulation of miR-508, thereby defining the stem-like/mesenchymal subtype and promoting stemness and metastasis in CRC (85).

Aberrant activation of the Hedgehog pathway is critical for maintaining the tumorigenic potential and stemness of CSCs. Members of the Gli family are key downstream factors in the Hedgehog pathway (86). Upregulation of LINC01106, which regulates Gli family members through a positive feedback loop, was found to promote the growth and stemness of CRC. Moreover, LINC01106 in the cytoplasm acts as a miR-449b-5p sponge to positively regulate the expression of the protein GLI family zinc finger 4 (Gli4). Fusion protein (FUS) is a transcription factor and an RBP associated with a variety of malignancies (87–89). LINC01106 in the nucleus recruits FUS to the Gli1 and Gli2 promoters, further activating their transcription. Because Gli2 is a transcriptional activator in the upstream promoter region of LINC01106 that promotes LINC01106 expression, Gli2 mediates the up-regulation of Gli1, Gli2 and Gli4 through LINC01106 to promote the growth and stemness of CRC (90).

The miRNAs miR-145 and miR-21 have been shown to synergistically regulate the proliferation and differentiation of CRCSCs (91, 92). The lncRNA CCAT2 was found to reduce the expression of miR-145 by inhibiting its maturation in colon cancer cells, accompanied by elevated miR-21, which in turn promotes the activities of CRCSCs. CCAT2 enriched in the nucleus inhibits the export of pre-miR-145 to the cytoplasm by blocking Dicer’s cleavage of pre-miR-145 and selectively blocks the maturation of miR-145 (93). These findings indicate that lncRNAs do not necessarily regulate miRNA expression through the ceRNA mechanism.

**Figure 1:**
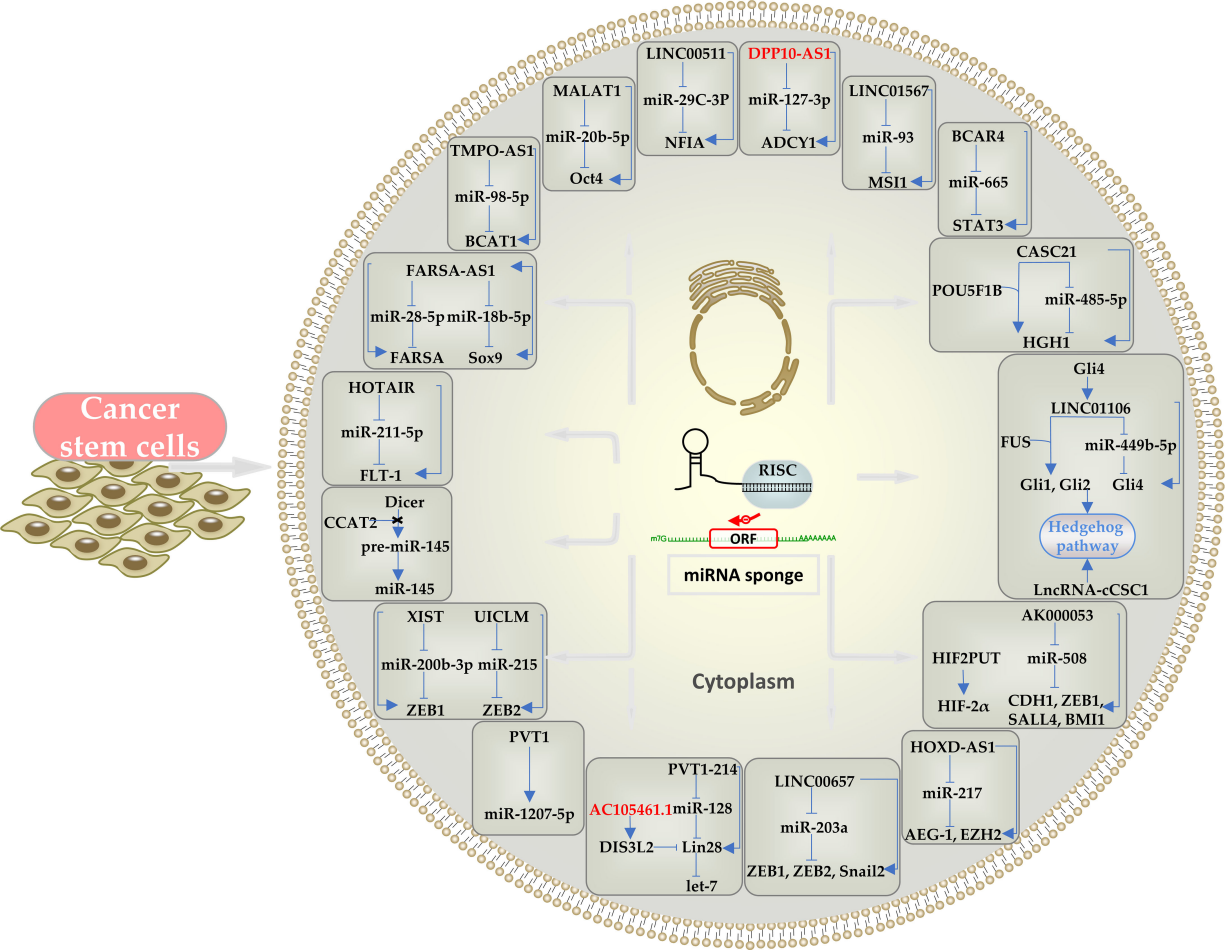
Schematic representation of the effects of cytoplasmic lncRNAs in CRCSCs through ceRNA mechanism and the downstream pathways and mediators involved. LncRNAs can act as a miRNA sponge. Oncogenic lncRNAs are indicated in black and tumor-suppressor lncRNAs in red.

The main effects of cytoplasmic lncRNAs in CRCSCs, acting through ceRNA and signaling pathways are summarized in **Table 1** and **Figure 1**. Because a single mRNA can be targeted by multiple miRNAs and a single miRNA can target multiple mRNAs, lncRNAs in CRC cells may be targeted by miRNAs other than those identified to date.

**Table 1:** LncRNAs functioning in CRCSCs.

LncRNAs		Function	CRCSCs subtype	Targets	Signaling pathway	effects	mechanism	Reference
**LINC00511**		Oncogene	Sox-2, Oct-4, CD44, Nanog	NFIA	LINC00511/miR-29c-3p/NFIA	Stemness, proliferation, metastasis	Post-transcriptional regulation	(54)
**PVT1-214**		Oncogene	Sphere formation	Lin28	PVT1-214/miR-128/Lin28/let-7	Stemness, proliferation, metastasis	Post-transcriptional regulation	(57)
**AC105461.1**		Suppressor	CD133, CD44	DIS3L2	AC105461.1/DIS3L2	Inhibition of stemness	NA	(60)
**PVT1**		Oncogene	CD44^+^CD166^−^, CD44, CD166, CD133	miR-1207-5p	Sox2/PVT1/miR-1207-5p	Stemness, incidence of ethnic differences	PVT1 is the host gene of miR-1207-5p, transcriptional regulation	(62)
**HOXD-AS1**		Oncogene	CD44, CD133, CD24, CD166, Oct4, LGR5, Sox2, Nanog	AEG-1, EZH2	HOXD-AS1/miR−217/AEG-1, EZH2	Stemness, proliferation, metastasis	Post-transcriptional regulation	(64)
**CASC21**		Oncogene	Sphere formation	HGH1	Cytoplasm: CASC21/miR-485-5p/HGH1	Stemness, proliferation, metastasis	Post-transcriptional regulation	(65)
					Nucleus: CASC21+POU5F1B/HGH1		Transcriptional regulation	
**TMPO-AS1**		Oncogene	Oct4, Nanog, Sox2	BCAT1	TMPO-AS1/miR-98-5p/BCAT1	Stemness, proliferation, apoptosis inhibition	Post-transcriptional regulation	(66)
**DPP10-AS1**		Suppressor	CD133^+^ cell sorting, CD44, LGR5, ALDH1	ADCY1	DPP10-AS1/miR-127-3p/ADCY1	Inhibition of proliferation, migration, invasion; promotion of apoptosis of CRCSCs	Post-transcriptional regulation	(67)
**TPTEP1**		Oncogene	spheroid cells, Oct4, Sox2, c-Myc, KLF4, Nanog	DDIT4, SULF1	TPTEP1/miR-148b-3p/DDIT4, SULF1	Stemness	Post-transcriptional regulation	(70)
**BCAR4**		Oncogene	ALDH^+^ cell sorting, Nanog, Oct4, Sox2, CD44, CD133, LGR5	STAT3	BCAR4/miR-665/STAT3	Stemness	Post-transcriptional regulation	(71)
**MALAT1**		Oncogene	CD133, CD44, Oct4, Nanog, Sox2, Notch1	Oct4	MALAT1/miR-20b-5p/Oct4	Stemness, cellular metabolism	Post-transcriptional regulation	(72)
**FARSA-AS1**		Oncogene	ALDH, CD133	Sox9, FARSA	Sox9/FARSA-AS1/miR-18b-5p/Sox9	Stemness, proliferation, metastasis	Post-transcriptional regulation	(73)
				FARSA-AS1/miR-28-5p/FARSA				
**LINC01567**		Oncogene	CD133^+^/CD166^+^/CD44^+^ cell sorting, MSI1, Oct-4, Sox2, ABCG2	MSI1	LINC01567/miR-93/MSI1	CRCSCs proliferation, metastasis	Post-transcriptional regulation	(75)
**XIST**		Oncogene	Nanog, Oct-4, Sox2, CD24, CD44, CD133, CD155, CD166	ZEB1	XIST/miR-200b-3p/ZEB1	Stemness, metastasis	Post-transcriptional regulation	(78)
**UICLM**		Oncogene	Nanog, Oct-4, Sox2, Notch1, ABCG2, CD24, CD44, CD133, CD155, CD166	ZEB2	UICLM/miR-215/ZEB2	Stemness, proliferation, liver metastasis	Post-transcriptional regulation	(79)
**LINC00657**		Oncogene	CD133^+^CD44^+^ cell sorting	ZEB1, ZEB2, Snail2	LINC00657/miR-203a/ZEB1, ZEB2, Snail2	CRCSCs invasion	Post-transcriptional regulation	(80)
**HIF2PUT**		Oncogene	Oct4, Sox2, CD44	HIF-2α	HIF2PUT/HIF-2α	Stemness	NA	(84)
**AK000053**		Oncogene	Nanog, c-Myc, SALL4, BMI1	CDH1, ZEB1, SALL4, BMI1	Hypoxia-inducible AK000053/miR-508/CDH1, ZEB1, SALL4, BMI1	Stemness, metastasis	Post-transcriptional regulation	(85)
**LINC01106**		Oncogene	Nanog, Oct4	Gli1, Gli2, Gli4	Cytoplasm: LINC01106/miR-449b-5p/Gli4	Stemness, proliferation, migration	Post-transcriptional regulation	(90)
					Nucleus: Gli2/LINC01106+FUS/Gli1, Gli2		Transcriptional regulation	
**CCAT2**		Oncogene	CD44, Sox2	miR-145	CCAT2+pre-miR-145	Stemness	NA	(93)
**NEAT1**		Oncogene	ALDH1, c-Myc, CD133, Sox2, Nanog, Oct4	ALDH1, c-Myc	NEAT1/acetylation of histones in the promoter region of ALDH1, c-Myc	Stemness, 5-Fu resistance	Histone modification regulation	(94)
**HOTAIR**		Oncogene	CD133^+^ cell sorting	FLT-1	HOTAIR/miR-211-5p/FLT-1	CRCSCs proliferation, metastasis	Post-transcriptional regulation	(95, 96)
**Hotair**		Oncogene	LGR5, c-Myc	NA	NF-κB/circulating Hotair in the form of exosomes/Wnt pathway	Intestinal stem and/or progenitor cells proliferation	NA	(97)
**HotairM1**		Suppressor	CD133^+^CD44^+^ cell sorting, Oct4, Sox2, Nanog	HOXA1	HotairM1/PRC2 complex/HOXA1/Nanog	Inhibition of self-renewal in CRC and uveal melanoma CSCs	Histone modification regulation	(98)
**Lnc34a**		Oncogene	CD133^+^CD44^+^ALDH1^+^ cell sorting	Notch1	Lnc34a/HDAC1+DNMT3a/miR-34a/Notch1	Initiation of asymmetric division of CRCSCs	DNA methylation, histone modification regulation	(99)
**B4GALT1-AS1**		Oncogene	ALDH1, Nanog, CD110, LGR5, CD44, CD133, EpCAM	YAP	B4GALT1-AS1+YAP	Stemness, metastasis	Transcriptional regulation	(100)
**LncGata6**		Oncogene	LGR5^+^ ISCs cell sorting	Ehf	LncGata6+NURF complex/Ehf/LGR4/5	Stemness of intestinal stem cells, intestinal tumorigenesis	Transcriptional regulation	(101)
**WiNTRLINC1**		Oncogene	ASCL2	ASCL2	ASCL2/WiNTRLINC1+β-catenin-TCF4/ASCL2	Stemness, proliferation, apoptosis inhibition	Transcriptional regulation	(102)
**RBM5-AS1(LUST)**		Oncogene	CD24^+^CD44^+^ cell sorting, CD133, CD166, ALDH1A1	SGK1, YAP1, MYC	RBM5-AS1(LUST)+β-catenin-TCF4/SGK1, YAP1, MYC	Stemness	Transcriptional regulation	(103)
**lncRNA-ATB**		Oncogene	Sphere formation	β-catenin	lncRNA-ATB/β-catenin	Stemness	Transcriptional regulation	(104)
**lincRNA-p21**		Suppressor	ALDH^+^ cell sorting, EpCAM, CD44, LGR5, Nanog, Oct4	β-catenin	miR-451/lincRNA-p21/β-catenin/PDK1/PDH	Inhibition of CRCSCs and their aerobic glycolysis (Warburg effect)	Transcriptional regulation	(105)
**LINC00525**		Oncogene	CD44, Sox2, Oct4	ELK3	LINC00525/miR-507/ELK3	Stemness, oxaliplatin resistance	Post-transcriptional regulation	(106)
**CRART16**		Oncogene	CD133, CD44	ERBB3	CRART16/miR-371a-5p/ERBB3	Stemness, Cetuximab resistance	Post-transcriptional regulation	(107)
**Linc00346**		Oncogene	Sphere formation	WBSCR22	Linc00346/miR-509-5p/WBSCR22	Stemness, 5-Fu resistance	Post-transcriptional regulation	(108)
**H19**		Oncogene	ALDH1, Nanog, Oct4, Sox2, CD44, CD133	β-catenin	Exosomal H19/miR-141/β-catenin	Stemness, oxaliplatin resistance	Post-transcriptional regulation	(109)
**TINCR**		Oncogene	Oct4, Sox2	TCF4	TINCR/miR-137/TCF4	Radiotherapy resistance	Post-transcriptional regulation	(110)
**LincRNA-ROR**		Oncogene	CD133^+^CD44^+^ cell sorting, Oct4, Sox2, Nanog	Oct4, Sox2, Nanog	LincRNA-ROR/miR-145/Oct4, Sox2, Nanog	Stemness, proliferation, cisplatin, paclitaxel resistance	Post-transcriptional regulation	(111)
**MIR600HG**		Suppressor	ALDH1A3, Sox2, CD44	ALDH1A3	MIR6S00HG/ALDH1A3	Inhibition of stemness, metastasis, oxaliplatin resistance	Post-transcriptional regulation	(112)
							Translation regulation	
**FENDRR**		Suppressor	spheroid cells, ALDH, Oct4, Sox2, KLF4	Sox2	FENDRR/Sox2	Inhibition of stemness, 5-Fu resistance	Post-transcriptional regulation	(113)
regulation	Translation regulation					Translation regulation		
**GAS5**		Oncogene	spheroid cells, Oct4, Sox2	NODAL	GAS5/NODAL pathway	Stemness, 5-Fu, adriamycin resistance	NA	(114)
**LncRNA-cCSC1**		Oncogene	CD133^+^CD44^+^ cell sorting	Hedgehog pathway	LncRNA-cCSC1/Hedgehog pathway	Stemness, 5-Fu resistance	NA	(115)
**Lnc273-31 and lnc273-34**		Oncogene	spheroid cells, Sox2, Oct4, Nanog, ALDH	Snail, ZEB1	P53-R273H mutation/lnc273-31, lnc273-34/Snail, ZEB1	Stemness, metastasis, oxaliplatin resistance	NA	(116)

NA, data not available.

### LncRNAs Regulate CRCSCs Through Epigenetic Modifications

**Figure 2:**
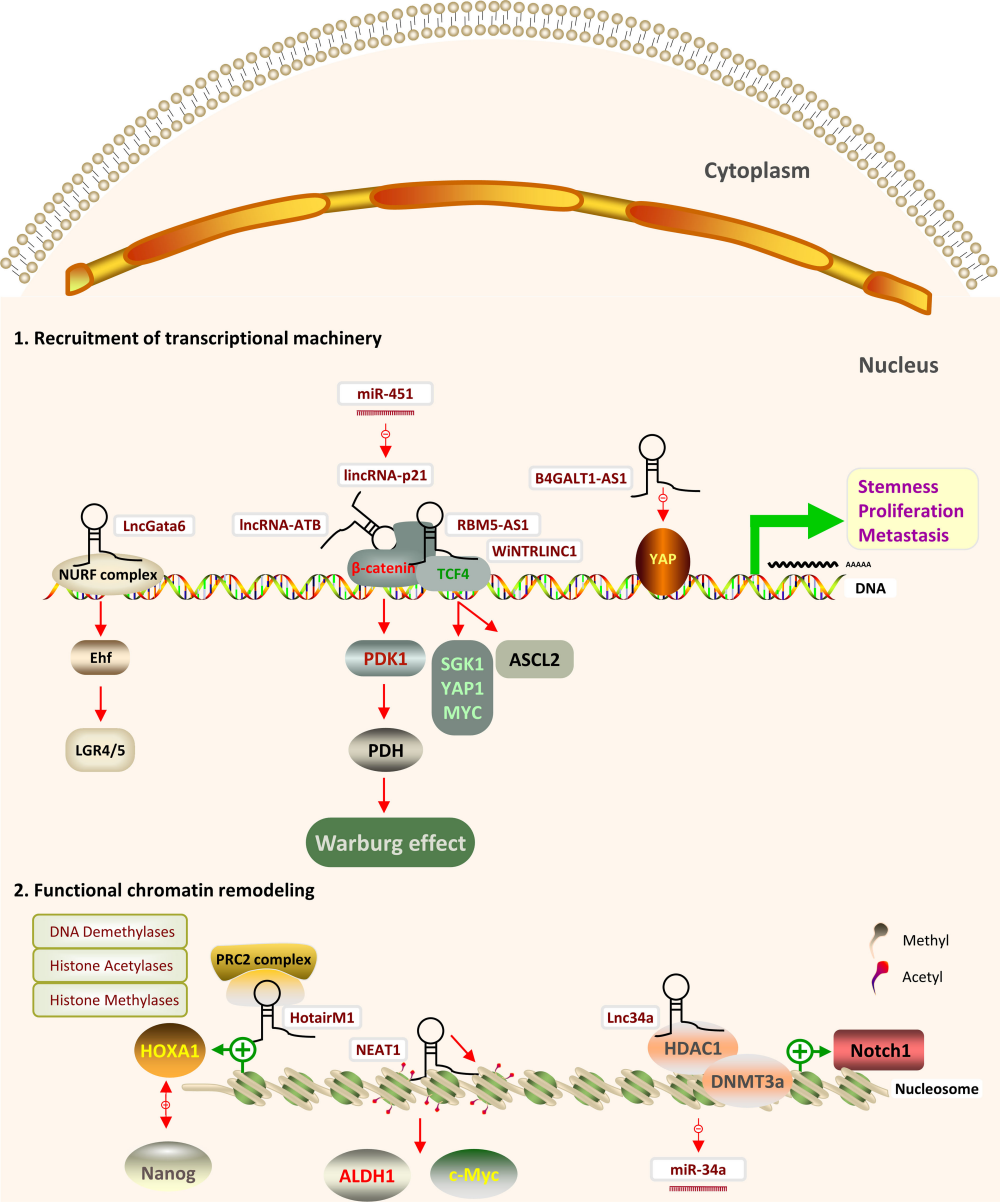
Schematic representation of the effects of nuclear lncRNAs through epigenetic modifications and transcription factors in CRCSCs and the downstream pathways and mediators involved. (1) LncRNAs can modulate transcription by transcription factors (TF) (2) LncRNAs can regulate DNA and histone modifying proteins by recruiting chromatin remodeling complexes.

Epigenetic modifications are stable genetic changes that affect gene expression and cellular phenotype without altering the coding gene sequence. These modifications mainly include changes in chromatin accessibility (chromatin remodeling), such as histone covalent modifications (e.g., acetylation, phosphorylation, methylation, ubiquitination, and SUMOization), DNA methylation and non-coding RNA regulation. The epigenetic pathogenesis of tumors is characterized primarily by abnormalities in DNA methylation and histone modifications in tumor cells (27). Epigenetic modifications regulate the differentiation and development of CSCs by altering gene transcription and expression. The status of DNA methylation and histone modifications of functionally important genes differ in undifferentiated CSCs and CSCs that have entered the differentiation process. This section of the review will summarize the functional involvement of lncRNAs in epigenetic modifications in CRCSCs (**Table 1**, **Figure 2**).

The highly expressed lncRNA NEAT1 has been found to promote CRC stemness and 5-fluorouracil (5-FU) resistance through a chromatin remodeling mechanism. ATAC-sequencing and other techniques showed that NEAT1 increased the acetylation of histones at ALDH1 and c-Myc promoters and enhanced the expression of these genes. These results not only identified a novel role for NEAT1, but may provide a new strategy for the treatment of 5-FU resistant CRC (94). The HOX transcript antisense RNA (HOTAIR) was shown to promote CRC stemness by targeting miR-211-5p to upregulate fms-like tyrosine kinase-1 (FLT-1) expression (95). Knockdown of HOTAIR significantly inhibited the proliferation, migration and invasion of CD133^+^ CRCSCs isolated by a magnetically activated cell sorting system (96). A sedentary lifestyle was observed to promote the release of Hotair in the form of exosomes from gluteal femoral fat, enhancing the proliferation of intestinal stem and/or progenitor cells and the development of CRC. NF-κB has been shown to promote Hotair transcription in adipose tissue, resulting in the increased secretion of exosomal Hotair, followed by its circulation in the blood and partial endocytosis by the intestines, ultimately promoting the proliferation and stemness of intestinal stem/progenitor cells by activating the Wnt pathway (97). Several isoforms of Hotair are also involved in tumor suppressor. For example, HotairM1, an antisense transcript of the HOXA1 gene in the homeobox genes (HOX) gene cluster, has been reported to be aberrantly expressed in a variety of tumors and is a key factor in tumorigenesis and progression. LncRNA HotairM1 depletion promotes the self-renewal of human CRC and uveal melanoma CSCs through the HOXA1-Nanog regulatory loop. Transcriptome sequencing of CRCSCs enriched in sphere-forming properties found that lncRNA HotairM1 was the most significantly downregulated lncRNA involved in the regulation of CSCs. HotairM1 recognizes and binds to the HOXA1 promoter and competitively inhibits the recruitment of SUZ12 and EZH2, components of the polycomb repressive complex2 (PRC2) in this region. Knockdown of HotairM1 resulted in the recruitment of SUZ12 and EZH2 to the HOXA1 promoter, enhancing H3K27 trimethylation. This, in turn, led to the epigenetic silencing of HOXA1, which promoted the acetylation of H3K27 at the Nanog enhancer site, resulting in increased Nanog expression. Increased Nanog expression can inhibit the acetylation of the HOXA1 enhancer site H3K27, further suppressing HOXA1 expression and forming a reciprocal regulatory loop that enhances CRC stemness (98).

Asymmetric cell division is a mode of proliferation unique to stem cells, generating self-renewing daughter stem cells and differentiated daughter cells to create cellular diversity (117). Interfering with asymmetric division can alter the balance of self-renewal and differentiation of cancer stem cells and affect tumor growth (118, 119). The Notch pathway is a key regulator of asymmetric division in many types of normal stem cells (120). Bu P et al. found that Notch pathway has been shown to regulate the fate of CRCSCs by altering the ratio of symmetric and asymmetric cell division through microRNAs. The distribution of miR-34a, which targets Notch1, has been found to determine the division pattern of CRCSCs. Early expression of high-level miR-34a in CRCSCs sequesters Notch1 mRNA, thus balancing progeny cell differentiation and self-renewal; whereas late expression of miR-34a induces an imbalance in CRCSCs, which undergo self-renewal (121). Lnc34a was found to initiate asymmetric division of CRCSCs by targeting miR-34a and triggering its spatial imbalance. Lnc34a, which is asymmetrically distributed during the division of CRCSCs, recruits histone deacetylase 1 (HDAC1) and DNA methyltransferase 3a (DNMT3a) through prohibitin-2 (PHB2). HDAC1 deacetylates histones, whereas DNMT3a methylates DNA in the promoter region of miR-34a, epigenetically silencing miR-34a expression and making miR-34a transcription independent of its upstream regulator, the p53 protein (99).

Additional studies are needed to determine the causes and consequences of these epigenetic changes that accompany different cancer cell states and to assess how pharmacological activation or inhibition of these epigenetic regulators can improve anticancer therapy.

### LncRNAs Regulate CRCSCs Through Transcription Factors

Transcription factors (TFs) are proteins in eukaryotic cells that assist in and regulate the transcription of mRNAs by RNA polymerase. Some core TFs are proteins encoded by oncogenes or tumor suppressor genes and are directly involved in target gene transcription during cell proliferation by binding to the cis-acting elements of the target genes (122). The following section provides a brief overview of some of the most important lncRNAs that act as TFs in CRCSCs (**Table 1**, **Figure 2**).

YAP/TAZ, a downstream executor of the Hippo pathway, has been shown to be a component of the β-catenin cytoplasmic destruction complex. Translocation of YAP/TAZ to the nucleus has been shown critical in cells activated by the Wnt/β-catenin signaling pathway (123). YAP plays a key role in the development of CSCs (124). For example, the lncRNA B4GALT1-AS1 was shown to promote CRC stemness and metastasis by recruiting YAP to the nucleus and enhancing YAP transcriptional activity (100). Intestinal epithelial cell development and differentiation are dependent on the concentration gradients of Wnt-BMP hedging signals in the intestinal microenvironment (7). Wnt signaling is a determinant of CRC stemness and controls essential stem cell genes such as LGR4 and LGR5 (125, 126). The nucleosome remodeling factor (NURF) complex plays an important role in regulating normal tissue and cancer stem cells (127, 128). LncRNAs induce carcinogenesis in normal intestinal stem cells (ISCs) by mediating the dysregulation of signaling pathways leading to uncontrolled cell proliferation and differentiation. Zhu P et al. verified that LncGata6, which is highly expressed in ISCs and CRCSCs, was shown to be involved in maintaining the stemness of ISCs and promoting intestinal tumorigenesis. Moreover, lncGata6-deficient intestinal epithelial cells failed to regenerate after damage. LncGata6 localized to the nucleus enhances LGR4/5 expression and activates the Wnt signaling pathway by binding to the Bptf subunit in the NURF complex, recruiting the NURF complex to the ETS homologous factor (Ehf) promoter region and promoting its transcription (101). GATA binding protein 6 (GATA6) is a transcription factor important in the expansion of CSCs and in the regulation of human CRC cell stemness, promoting the expression of LGR5 in CSCs (129). The expression profiles of lncGata6 and the nearby protein-encoding gene Gata6 differ, suggesting that these two molecules may play different roles in different mouse tissues and that the deletion of lncGata6 did not affect Gata6 expression (101). Achaete scute-like 2 (ASCL2) is an important stemness transcription factor that controls ISCs in response to Wnt signals (130). ChIP-seq of RNA polymerase II revealed that the Wnt-regulated lincRNA1 (WiNTRLINC1), which is about 60 kb away from the ASCL2 gene, is one of the direct targets of β-catenin/TCF4 in CRC. WiNTRLINC1 promotes the cyclization of regulatory elements by interacting with β-catenin/TCF4, thereby activating transcription of the ASCL2 gene in a cis manner. The regulatory Wnt-WiNTRLINC1-ASCL2-stemness network was further enhanced by ASCL2-induced transcriptional activation of WiNTRLINC1 (102). Although high expression of WiNTRLINC1 in CRC is associated with increased metastatic potential and poorer prognosis, Snail1, a master regulator of EMT, was found to reduce EMT-related proliferation by directly repressing the stemness-related genes MYB and WiNTRLINC1, while abrogating the stemness characteristics of CRC cells (131). Thus, the relationship between EMT and stemness varies among different tumor types.

The lncRNA RBM5-AS1 (LUST) has been shown to promote stemness in CRC by interacting with β-catenin and promoting its interaction with the TCF4 complex at target genes. This, in turn activates the Wnt signaling pathway and downstream SGK1, YAP1 and MYC (103). At the same time, lncRNA-ATB promotes CRC stemness by enhancing the transcriptional activity of β-catenin (104). LncRNA-ATB was shown to promote CRC metastasis by inhibiting E-cadherin (132). In contrast, TGF-β-activated lncRNA-ATB was found to upregulate ZEB1 and ZEB2 by competitively binding to members of the miR-200 family and to upregulate IL-11 expression in an autocrine manner by binding to and stabilizing IL-11 mRNA, activating the STAT3 signaling pathway and promoting the invasive metastatic cascade in hepatocellular carcinoma (133). Taken together, these findings suggest that lncRNA-ATB may also be present in CRC, promoting metastasis through multiple pathways, including CSCs.

There are emerging evidences that CSCs display elevated glycolytic metabolism, as compared to their differentiated counterparts. Reversal of glycolysis to oxidative phosphorylation was associated with impairment in the proliferation and stem cell properties of CSCs (134–136). The expression profile of lincRNA-p21, a direct transcriptional target of the tumor suppressor p53, was found to be low in CRCSCs. Overexpression of lincRNA-p21 inhibited aerobic glycolysis in ALDH^+^ CSCs by suppressing the β-catenin/pyruvate dehydrogenase kinase 1 (PDK1)/pyruvate dehydrogenase (PDH) signaling axis. This reversal may be an effective approach to shift the glucose metabolic program in CSCs and may contribute to the elimination of CSCs from cancer tissues (105). MiR-451 has been reported to negatively regulate self-renewal, tumorigenicity and drug resistance in CRCSCs (137). To avoid the off-target effects of lincRNA-p21, Ad-lnc-p21-MREs were constructed by integrating the miRNA responsive elements (MREs) of miR-451 into adenoviral vectors expressing lincRNA-p21, allowing lincRNA-p21 to be specifically and efficiently expressed in ALDH^+^ CSCs (105). Gene therapy regulated by MREs is a promising strategy for targeted treatment of CSCs, highlighting the need to evaluate the safety of engineered adenoviral vectors for use in gene therapy.

### LncRNAs Mediate Chemoradiation Resistance in Colorectal Cancer Through CRCSCs

**Figure 3:**
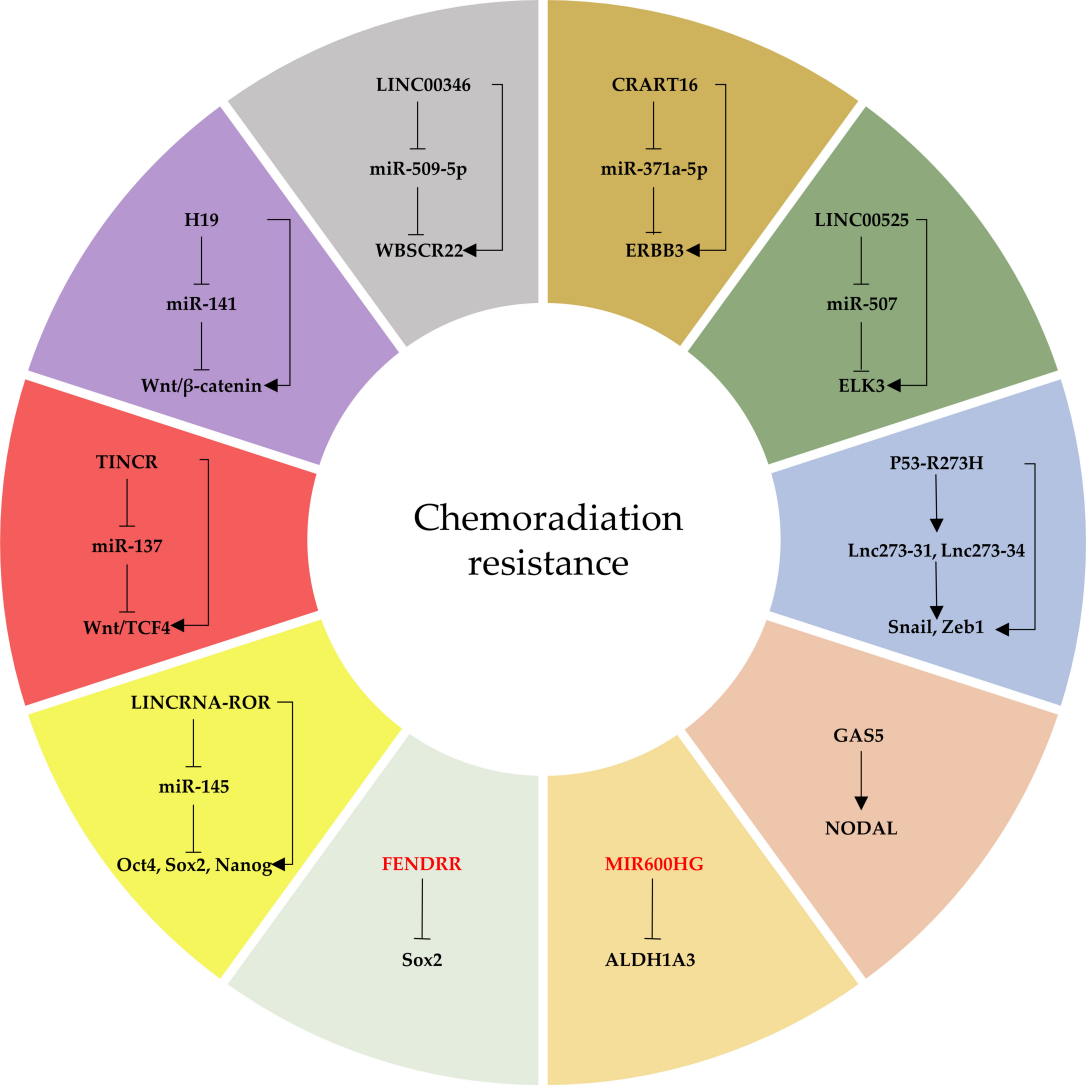
The network of lncRNAs that regulate CRCSC-mediated chemoradiation resistance. Oncogenic lncRNAs are indicated in black and tumor-suppressor lncRNAs in red.

Based on the drug resistance mechanism of CSCs, three modalities are key to treatment of CSCs: directly targeted killing, induction of differentiation and promotion of proliferative phase (35). LncRNAs affect tumor resistance to multiple drugs, including pentafluorouracil, oxaliplatin and cetuximab, through the stemness pathway, and may become novel targets for the clinical treatment of CSCs (**Figure 3**).

ELK3 has been associated with the development and progression of different types of cancer and is a key factor in resistance to chemotherapy (138, 139). LINC00525 was shown to mediate the upregulation of ELK3 expression through miR-507, promoting CRC stemness and resistance to oxaliplatin (106). V-Erb-B2 erythroblastic leukemia viral oncogene homolog 3 (ERBB3), an important member of the human epidermal receptors (HER), is involved in signaling crosstalk with EGFR (140). This crosstalk is part of the mechanism by which tumors bypass EGFR TKIs and can activate the downstream MAPK pathway, thereby contributing to cetuximab resistance in CRC (141, 142). A new lncRNA CRART16 was identified by targeting miR-371a-5p to increase ERBB3 expression, thereby promoting CRC stemness and cetuximab resistance (107). Linc00346 competes with WBSCR22 for miR-509-5p binding sites, thereby regulating the phenotype of CRCSCs (108). WBSCR22 was found to promote oxaliplatin resistance in CRC by targeting miR-146b-5p (143). It may well be speculated that Linc00346 may also be involved in the ability of WBSCR22 to regulate CRCSCs, which is also associated with oxaliplatin resistance. Cancer-associated fibroblasts (CAFs) transfer exosomal lncRNA H19 into cancer cells. H19 has been found to activate the β-catenin pathway in CRC cells by acting as a competitive endogenous RNA sponge for miR-141, promoting stemness and chemoresistance to oxaliplatin (109). TCF4 is a key protein in the Wnt pathway of CSCs (144). LncRNA TINCR was shown to promote radiotherapy resistance in CRC cells by affecting the TINCR/miR-137/TCF4 axis. The stemness genes Oct4 and Sox2 are highly expressed in the radiotherapy-resistant SW620R CRC cell line, and knockdown of TINCR reduced the sphere-forming ability and stemness factor expression in these cells while restoring their sensitivity to radiotherapy (110). In another study, lincRNA-ROR was found to affect the biological properties of CRCSCs, regulating their proliferation and sensitivity to cisplatin and paclitaxel, by targeting miR-145 to upregulate the expression of Oct4, Sox2 and Nanog (111).

MIR600HG was observed to inhibit stemness, metastasis and oxaliplatin resistance in CRC by targeting ALDH1A3, and overexpression of MIR600HG combined with oxaliplatin treatment was found to inhibit tumor recurrence. The MIR600HG seed sequence affects the stability and translational activity of ALDH1A3 mRNA by binding to its 3’UTR, thereby inhibiting ALDH1A3 mRNA and protein expression (112). Some lncRNAs processed into miRNAs were found to repress the expression of their target genes (145). We speculate that miRNAs derived from MIR600HG may target and regulate the expression of ALDH1A3 mRNA. In addition, the lncRNA FENDRR was found to directly interact with the 3’UTR of Sox2 mRNA, reducing its stability and inhibiting the expression of Sox2 and CSC-like traits in CRC cells (113).

NODAL signaling plays an important role in regulating stemness and inducing chemoresistance in CSCs (146). The lncRNA GAS5 (growth-arrest-specific transcript 5) was found to promote CRC stemness and resistance to 5-FU and adriamycin in a NODAL-dependent manner. Interestingly, overexpression of GAS5 sensitized HCT116 cells to 5-FU and adriamycin, in contrast to the effects observed in CSCs derived from HCT116 cells (114). The novel oncogenic lncRNA-cCSC1 was found to promote the properties of CRCSCs through activation of the Hedgehog signaling pathway, enhancing 5-FU resistance. Although the expression of PCNA expression was substantially reduced in xenograft tumor tissues expressing shlncRNA-cCSC1 (115), it may be difficult to distinguish differences in the proliferation of liver CSCs and non-CSCs *via* Ki67 staining (147). Thus, the self-renewal properties of different cancer types may differ. TP53 is one of the most frequently mutated genes in all types of cancer, being present in over 60% of CRCs. The three key mutated loci in p53 are R175H, R248W and R273H, but it is unclear whether mutant p53-regulated lncRNAs are associated with CSCs (148). Cells bearing the p53-R273H mutation exhibited more characteristics of CSCs than cells bearing the p53-R175H and p53-R248W mutations. The P53-R273H mutation enhanced Snail and ZEB1 expression and the EMT process by upregulating the expression of lnc273-31 and lnc273-34, thus enhancing CRC stemness. RNA-seq analysis of spheroid cells with endogenous p53 point mutations induced using a somatic knock-in method to establish a network of p53-R273H-regulated lncRNAs showed that the parent gene at the site of the lncRNA has a P53 binding site (116).

The role of some lncRNAs in CRCSCs is outlined below and summarized in **Table 1**. The discovery of ncRNAs has added a new dimension to the understanding of cancer development and treatment, by providing a window into the effects of ncRNAs throughout the rest of the genome.

## Research Prospect and Clinical Value of LncRNAs Targeting CRCSCs

The concept of CSCs has not only deepened understanding of the mechanisms of tumorigenesis, but has suggested new ideas for research on tumor treatment. Further research on CSCs and their abnormal microenvironments may enable the identification of new drug targets for tumor therapy and the development of more effective treatments. However, several aspects related to the functions and regulatory mechanisms of CSCs must still be addressed.

1) Studies have demonstrated the existence of multiple types of CRCSCs that play different roles in tumor maintenance and metastasis formation (149). This suggests the need for additional studies on levels of cellular heterogeneity among CSCs and cellular hierarchies in CRC. CSCs expressing different stemness markers belong to different subpopulations and have different characteristics, suggesting that lncRNAs may have different functions in these cells. Identification of characteristic biomarkers of CSCs is the key to initiating studies of the mechanisms that regulate CSCs. Isolating and identifying CSCs are challenging without identifying CSC-specific markers. In addition, differentially expressed lncRNAs in CSCs are not specific for CSCs, and interfering with lncRNAs in CSCs can also simultaneously alter tumor cells. Standard indirect methods of identifying CSCs include *in vitro* tumor sphere formation and *in vivo* limiting-dilution tumorigenicity assays in immunocompromised mice (16). The major limitation of this method is partly associated with difficulties in the ability to distinguish between CSC and non-CSC populations of cancer cells. In addition, differences between the microenvironments of the original and transplanted tumors may alter CSC functions. Thus, the identification and isolation of CSCs remain unclear due to the lack of unique isolation and identification methods and the complex biological properties of these cells.

Genetic-lineage tracing enables the identification and study of stem cells in solid tissues *in situ* while avoiding mechanical perturbation. This technique critically relies on the identification of individual marker genes, for example by enabling the stable activation of the reporter gene for LGR5 in the target cell population, resulting in the labeling of the target cells. Importantly, stable reporter gene expression is maintained in all daughter cells of the marked cell. Stemness potential is evaluated by determining the persistence, size, and composition of cell clones generated over time (150). The best current approaches to obtain CSCs depend on the use of a variety of cell surface markers, including the use of FACS technology to enrich for CSCs from primary tumor tissue and secondary sphere formation by digestion of cells enriched in suspension. Thus, precise characterization of a given CSC population requires a multifaceted approach based on tumor-specific markers combined with other analyses, such as functional assays, to establish a distinctive phenotypic profile. To validate the optimal CSC markers, specific combinations must be assessed in large patient populations, and the sensitivity and specificity of these markers must be tested by calculating areas under receiver operating characteristic (ROC) curves. Transcriptional and proteomic profiling of cells with established CSC properties should enhance our knowledge of these cells and provide such markers.

2) Determining whether CSCs are the origin of gene mutations during colorectal carcinogenesis is key to the prevention, diagnosis and treatment of CRC. To date, the cellular origin of each tumor-associated mutation, whether stem cells, transit amplifying (TA) cells, or normal tissue cells, remains unclear (151). CRCs originating from different cell types will differ in cell-specific mutated genes. These cells will also differ in specific tumor markers for pre-cancer diagnosis and loci for clinical intervention and targeted therapy. Resolution of the cellular origin of tumor-specific gene mutations will help in complete tracking of tumor cell lineage. For example, determination of their origin will clarify whether all tumor cells are derived from CSCs, as well as helping to determine patient prognosis at an early stage, including the ability to predict metastasis, recurrence, overall survival and the effect of treatment. In depth studies are therefore required to determine the role of CSCs in tumorigenesis and development.

Almost all CRC cells are derived from intestinal epithelial cells, and the continuous renewal capacity of intestinal epithelium is mainly maintained by intestinal stem cells in the glandular fossa. The microenvironment in which stem cells are located and the signaling factors regulating stem cells play very critical roles in maintaining stem cell proliferation, differentiation and intestinal epithelial homeostasis (152, 153). To date, the most studied intestinal stem cells are LGR4/5 positive stem cells (154). Because large numbers of CSCs originate from the oncogenic transformation of intestinal stem cells, determining the regulation of the proliferation and differentiation of intestinal stem cells is important in determining the pathogenesis of CRC.

Currently, the treatment of CRC consists of surgical resection supplemented by radiotherapy, chemotherapy and targeted therapy. The most frequently used chemotherapy regimen consists of a combination of 5-FU, folinic acid (LV), and oxaliplatin (mFOLFOX6 regimen). The efficacy of clinical treatment of CRC is limited by drug resistance, susceptibility to metastasis, low differentiation, high malignancy, and poor prognosis (155, 156). Drug resistance is a major challenge, and understanding the mechanisms of resistance is essential for the development of novel drugs for CRC treatment. CSCs are highly resistant to common anticancer drugs, due to their ability to self-renew, as well as their pluripotency, angiogenic potential, immune escape, symmetric and asymmetric division, and the presence of multiple DNA repair mechanisms. Conventional radiotherapy can only kill tumor cells and stromal cells, while CSCs are preserved. CSCs have the ability of self-renewal and multidirectional differentiation, with small numbers continuously proliferating and differentiating into tumor cells, resulting in tumor recurrence and metastasis (157). Combinations of chemotherapeutic drugs that selectively target CSCs and conventional radiotherapy, whether in neoadjuvant or adjuvant setting, may result in complete tumor eradication.

In addition, CSCs are frequently characterized by high expression of immune checkpoint proteins. The interactions of CSCs with various components of the tumor immune microenvironment (TME), including TAMs, MDSCs, DCs, and Tregs, can result in CSCs evading detection by the immune system. Simultaneously, CSCs can drive tumorigenesis and disease progression by regulating the balance of pro- and anti-tumor immune cell activity in the TME. The differences in the roles of shared signaling pathways in CSCs and immune cells limit the ability to administer treatments that inhibit these cascades. Thus, the use of drugs that target these pathways in CSCs must consider their off-target effects on immune cells (158, 159). Preclinical models and clinical trials evaluating the effects of these drugs on CSCs should also evaluate the effects of these drugs on host immune cells. Multiple approaches to target the interactions between CSCs and the immune system are being actively pursued, and a variety of immunotherapies targeting CSCs are currently in clinical development. These experimental therapeutic strategies include efforts to stimulate tumour- specific T cells, alter the immunosuppressive TME and target CSCs surface markers using antibody- based treatments (160, 161). Increased understanding of the interactions of CSCs with the immune system has laid the foundation for exploring these complex interactions and developing targeted therapeutic approaches. However, the optimal timing, sequence, and combination of these CSC-specific immunotherapies require further investigation.

Because of the multifaceted roles of lncRNAs on CRCSCs described above, our further analyses of lncRNAs are intended to provide a theoretical basis for clinical treatment of CRC and for determining their mechanism of action.

LncRNAs can be released by tumor cells into the blood as naked exosomes or vesicles (162, 163). The high specificity of methods to detect lncRNAs, as well as the tissue-specificity of these lncRNAs, will enable the use of circulating or secreted lncRNAs in body fluids (e.g., plasma or urine) as biomarkers for CRC diagnosis, prognosis, and response to treatment. These assays will provide an ideal non-invasive method, avoiding the need for tumor tissue biopsy in patients.

RNA-based therapeutic approaches offer advantages and disadvantages when compared with small molecules and biologic agents. The primary advantage is that RNA drugs are based on nucleotide hybridization. Thus, the design of an agent based on a specific sequence can allow the target to be approached easily and specifically. In contrast, small molecules and biologic agents require longer and more laborious screening or a structure-based design approach. In addition, RNA-based agents can easily target multiple RNAs. These approaches, however, also have disadvantages, such as the need to consider and optimize RNA-based drug delivery methods. Oral formulation of RNA-based therapeutics is more difficult than small molecules and biologics. Also, RNA-based drugs have been associated with immune-related toxicity and other adverse events (164). Inhibition of lncRNAs *in vivo* usually requires the design of oligonucleotides targeting lncRNAs and a delivery method. Methods such as antisense oligonucleotide (ASO) technology can inhibit lncRNAs, leading to their RNase H-dependent degradation (165). The stability and affinity of RNA drugs *in vivo* may be improved by chemical modifications, such as lock nucleic acid modifications (LNA). At present, liposome-based nanocarriers are the most established method for the *in vivo* delivery of RNA drugs (166).

Research on lncRNAs is still mainly at the theoretical level and has not yet been approved for clinical use in the treatment of CSCs. However, several clinical trials of monotherapy and combination therapy have been performed to assess the safety and efficacy of treatments that target CSC pathways. Identification of additional key lncRNAs that are involved in CRCSCs and their regulatory mechanisms are fundamental to improving the efficacy of new anticancer therapies. First, transcriptome microarray analysis of CSC subpopulations sorted from cell lines and primary cells by CSC markers and genome-scale activation screening may be the most feasible methods of determining the expression profile of lncRNAs in CRCSCs (167). Second, guilt-by-association (GBA) analysis has proposed that the function of a lncRNA can be inferred from the known biological functions of protein-coding genes (PCGs) with which it is co-expressed (168). Therefore, lncRNAs that are significantly associated with the expression of stemness genes should be studied in depth. Third, most cancer-related lncRNAs have the same expression patterns and biological functions, independent of the type of cancer. Thus, stemness-associated lncRNAs identified in normal stem cells or other CSCs can lead to the identification of novel lncRNAs in CRCSCs. Fourth, the ceRNA mechanism has suggested that lncRNAs that bind to known stemness miRNAs can regulate CSCs. Fifth, drug resistance-associated lncRNAs also have the potential to modulate CSCs.

Research on the lncRNA regulation of CSCs suggests that mechanisms rarely reported in CRCSCs, such as lncRNA regulation at the translational level or binding to DNA, are worth exploring in the future. In addition, highly conserved lncRNAs may be more likely to be functional. Notably, the hallmarks of cancer, such as genome instability and mutation, reprogramming energy metabolism, tumor-promoting inflammation, and evading immune destruction, are also applicable to CSCs, studies of these processes are important development directions of CSCs. The current availability of data on the expression of thousands of lncRNAs across CRCSCs can enable the identification of new lncRNAs.

## Conclusions

In summary, lncRNAs play an important regulatory role in the biological behavior of CRCSCs, such as self-renewal, proliferation, differentiation, metastasis and chemoradiation resistance, by regulating the expression of molecules related to various signaling pathways, pluripotent stem cell factors and other stemness genes. LncRNAs may have broad clinical applications, not only as biomarkers for diagnosis, staging and prognosis of patients with CRC, but also as targets for precision tumor treatment, providing new possibilities for targeted killing of CSCs. Targeting CSCs requires in-depth understanding of the biology, genealogical relationships, cellular functions and signaling mechanisms of CSCs and their derivatives in homeostasis and disease. LncRNA-based studies will contribute to a deeper understanding of the biology of CSCs.

However, many pressing issues remain to be addressed. First, the actual regulatory effects of lncRNAs in cells or animals are difficult to replicate in the complex human environment, requiring confirmation of the up- or down-regulation of lncRNAs in cancer. Second, because related research on lncRNAs is still in its initial stage, studies are required to identify factors that trigger their dysregulation. Moreover, additional efforts are required to determine the wide range of regulatory mechanisms and the diversity of downstream pathways of lncRNAs. Despite many challenges, a better understanding of the interactions between CSCs and lncRNAs may be the key to opening a new era of oncology therapy that is associated with a reduced propensity to develop drug resistance and enhanced anti-metastatic activity, which can ultimately improve patient prognosis.

## Author Contributions

Conceptualization, GW and NW. Methodology, QZ. Writing—original draft preparation, BF. Writing—review and editing, BF. All authors have read and agreed to the published version of the manuscript.

## Funding

The APC was funded by National Natural Science Foundation of China, grant number 81702867; Project of Wu Jieping Medical Foundation, grant number 320.6750.15183.

## Conflict of Interest

The authors declare that the research was conducted in the absence of any commercial or financial relationships that could be construed as a potential conflict of interest.

## Publisher’s Note

All claims expressed in this article are solely those of the authors and do not necessarily represent those of their affiliated organizations, or those of the publisher, the editors and the reviewers. Any product that may be evaluated in this article, or claim that may be made by its manufacturer, is not guaranteed or endorsed by the publisher.
